# Density of coral larvae can influence settlement, post-settlement colony abundance and coral cover in larval restoration

**DOI:** 10.1038/s41598-020-62366-4

**Published:** 2020-03-26

**Authors:** Kerry A. Cameron, Peter L. Harrison

**Affiliations:** Marine Ecology Research Centre, School of Environment, Science and Engineering, Southern Cross University, Lismore, New South Wales 2480 Australia

**Keywords:** Behavioural ecology, Conservation biology, Restoration ecology, Marine biology

## Abstract

Successful recruitment of new individuals is essential for recovery of degraded coral reefs. Enhancing supply of coral larvae increases initial settlement, however post-settlement survival can be influenced by density-dependent processes. We investigated the influence of larval density on settlement, colony abundance and growth to 24 months for *Acropora tenuis* in the north-western Philippines, to determine whether larval supply can be optimised to maximise successful recruitment. Thirty different densities of coral larvae were enclosed for five days around settlement tiles and highest total settlement occurred on tiles with highest larval densities. After 12 months, however, colony abundance and coral cover was lower on high density tiles (supplied with ~2,500–5,000 larvae) than tiles supplied with ~1,000–2,000 larvae. Coral cover at 24 months remained highest on tiles supplied with ~1,000–2,500 larvae. Larval density influenced larval substratum selection, with proportionally fewer larvae settling in typically preferred locations as density increased. We conclude that larval density can influence post-settlement colony abundance and coral cover to 12 months, with coral cover trends persisting to 24 months. We show that optimising larval densities can maximise coral recruitment and growth, however oversupply of larvae at very high densities can have negative outcomes for larval restoration.

## Introduction

Coral reefs are globally significant ecosystems that are declining from cumulative threats including climate change, destructive fishing practices, pollution and poor water quality^1,2^. In many regions, significant areas of coral reef habitat have been lost^3^, while in others the surviving corals are sparsely distributed and unable to recover within reprieve periods between significant disturbance events^4,5^. These accelerating global declines require increased conservation and management action to protect coral reefs, including development of innovative measures for active coral reef restoration^6–9^.

Successful recruitment of new corals is a critical ecological process for the maintenance and recovery of corals and reefs^5,10–12^. With some exceptions^13^, coral populations are often considered demographically open and the addition of new individuals is largely dependent on the supply of pelagic coral larvae from other reefs^14^, followed by suitable ecological conditions for settlement and post-settlement survival and growth^10,15^. Early trials to improve coral recruitment by enhancing larval supply found that providing high densities of mass-cultured larvae to *in situ* artificial reef substrata led to significantly increased coral settlement compared with settlement from natural larval supply at both Coral Bay, Western Australia^16^, and in Palau, Micronesia^17^. The long-term effects of enhancing larval supply were not monitored for Coral Bay, however, the experiment in Palau was monitored for 13 months post-settlement and found the positive effect of enhanced larval supply on recruitment did not persist beyond 30 weeks. This was attributed to high rates of ongoing background recruitment from natural larval supply within the Palau reef system, as well as high post-settlement mortality, which the authors considered could have been related to initial settlement density^17^. Overall, the Palau study concluded that provision of high densities of coral larvae was not an effective method for reef restoration, although noted that larval restoration may have a role at sites with “very low background recruitment”^17^. This was subsequently tested in northwestern Luzon in the Philippines, at a degraded coral reef with negligible natural recruitment^18^. High densities of mass-cultured coral larvae were supplied directly onto the degraded reef substrata under temporary enclosures of fine mesh, leading to significantly greater settlement than adjacent control sites where only natural larval supply was available. In contrast to the earlier work in Palau^17^, growth and survival of juvenile corals in treatment sites remained significantly higher than in control sites over multiple years^18^. Most corals attained sexual maturity after three years and re-established a reproductive population of foundation corals. This indicates that while enhancing larval supply has shown little impact on reefs with abundant natural supply^17^, larval restoration can be an effective method on degraded reefs where natural recruitment is limited^18^.

Methods for harvesting coral spawn and rearing competent coral larvae are well established and research into scaling up gamete collection and larval rearing processes is ongoing^19,20^. While this occurs, determining the optimal densities of coral larvae to supply to degraded reefs to maximise successful recruitment will assist effective distribution of the reared larvae. Positive relationships have been observed between the abundance of coral larvae supplied and numbers of initial settlers in larval densities examined to date^12,16,21^. However, space can be a limited resource for both initial settlement and subsequent growth of the sessile juvenile coral colonies^22^ and density-dependent relationships between settlement and recruitment have been observed in early life-history stages of *Acropora* species, an important early-colonising reef-building group^12,17^. Previous studies have reported highest rates of post-settlement mortality occurring in the highest settler densities investigated after one month^12,21^ and between five weeks to 30 weeks^17^. However, the effect of a wide range of initial larval densities on settlement through to longer-term post-settlement survival and recruitment has not been investigated.

Mortality of coral spat within six months of settlement is naturally very high^18,23,24^, therefore improving survival through this period is a key focus of coral reef restoration research^11,25^. Post-settlement mortality can result from predation, competition, overgrowth, limited energy and sedimentation^10,17,18,21,26,27^, with complex ecological trade-offs operating in response to multiple biophysical interactions throughout early coral ontogeny^15^. Structural interventions, such as cages to shelter newly settled recruits^11,25,28^, can increase post-settlement survival to varying degrees by reducing predation and sedimentation, although initial benefits may be lost over time as macroalgal growth within structural shelters can reduce the growth and survival of coral recruits^15,29,30^. In the absence of structural interventions, the potential impacts of predation and sedimentation on post-settlement survival are influenced by substratum selection choices made by each coral larva at the point of settlement^10,31,32^. Larvae that settle in sheltered positions with good light availability have a greater likelihood of survival than larvae settled in exposed or heavily shaded positions^33^. Larval preference for settlement positions with these ecological characteristics is evident from previous studies where high proportions of recruits were found on the vertical edges or cryptic undersides of flat settlement substrata^34–36^. However, where available space in these preferred positions is limited, ecological trade-offs between aggregated and solitary settlement may influence larval substratum selection. When coral larvae settle in aggregations, there are risks of increased competition for food resources (including for photosymbiont microalgae), disease and overgrowth by neighbours^21,37^. However, there is also potential for close neighbours to fuse in early life-history stages, which can lead to improved survival and rapid growth compared to non-fused colonies^38,39^. Investigating the relationship between larval density and substratum selection can provide insights into whether larval density may also influence settlement choices that impact longer term survival of coral recruits.

In this study, we extend the knowledge generated in previous larval density research by investigating coral settlement and abundance outcomes from a greater range of larval densities and over a longer period than has been examined before. Our objective was to determine the relationship between larval density and initial settlement, juvenile colony persistence and coral growth. We show that optimal densities for larval supply can be determined to maximise initial settlement and subsequent contribution to coral cover, which need to be considered when planning larval restoration of degraded coral reefs.

## Methods

### Experimental overview

An *in situ* field experiment was conducted over two years to examine the influence of larval density on initial settlement and post-settlement persistence, coral abundance and growth. Using a regression design, we tested 30 different larval densities of *Acropora tenuis* in a degraded reef environment. Larvae were initially confined within 30 individual flow-through enclosures each containing a pre-conditioned settlement tile, during the settlement period^34,40^. The containers were then removed and the tiles and settled spat exposed to the open reef environment. The branching coral species, *A. tenuis*, was selected due to its ecological significance within the study region, and because spawned gametes and larvae were readily available^18^.

### Site location and preparation

This study was conducted at Magsaysay Reef in the Lingayen Gulf, Anda, Pangasinan, Northern Luzon, the Philippines (16°19′36″N, 120°02′01″E). The reef site was characterised by low mean live scleractinian coral cover (~15%) and high mean cover of macroalgae, turf communities, sponges and soft corals (~57%)^18^, and encircled a small sand patch at 2–3 m depth.

Coral settlement and persistence of juvenile colonies were monitored on biologically conditioned natural settlement tiles. To mimic natural reef substratum as closely as possible while still allowing for periodic *ex situ* monitoring under microscopes, 30 tiles were cut from the skeletons of dead tabulate *Acropora* species collected from a nearby rubble zone^18^. The irregular surfaces provided a range of natural microhabitats, and each tile was fixed 0.5–2 cm above the substratum to provide the full range of surface orientation available on a natural reef, i.e., exposed upper surfaces, vertical surfaces, and shaded downward facing surfaces (see Supplementary Fig. S1). While there was some natural variation between tiles, average dimensions were 10 × 10 × 2.5 cm (~300 cm^−2^ surface area). Prior to the experiment, settlement tiles were first conditioned for 6 weeks in aquaculture tanks with flow-through seawater and aeration to acquire initial biofilms at the Bolinao Marine Laboratory (BML), of the University of the Philippines Marine Science Institute. Then on 30 March 2017 the tiles were transferred to the reef site at Magsaysay Reef for an additional ~8 weeks conditioning to further develop biofilms *in situ*. Tiles were attached to a threaded steel rod projecting from a stainless steel plate fixed directly to the substratum^41^ (see Supplementary Fig. S1). A coded stainless steel tag was attached to each tile and stainless steel plate, to ensure tiles could be returned to the same location and relocated on the steel posts in the same orientation after monitoring.

### Gamete collection and larval culture

Coral cover at Magsaysay Reef is very low, however there are several discrete patches where colonies of sexually mature *A. tenuis* are present, following previous larval restoration trials^18^. These patches are approximately 50 m from the experimental site for this study. Field sampling of the *A. tenuis* colonies on 5 May 2017 revealed dark pink mature eggs, indicating imminent spawning^42^, with spawning occurring during a fierce electrical storm between 18:30 h and 19:00 h on 10 May 2017, the night of the full moon. During this time, spawn collection nets made from 150 μm plankton mesh were placed over 30 spawning colonies by divers (see Supplementary Fig. S2). Spawned egg-sperm bundles were positively buoyant and collected in a jar attached to the top of each net. Jars were sealed *in situ* after spawning and gametes from each colony were kept separated during transport to BML, where all gametes were transferred into a large polyethylene container (60 L Nally bin) with ~40 L of 1 µm filtered seawater for fertilisation. Contribution to the larval pool from each colony was roughly equivalent as netted colonies were similar in size and most of the spawned gamete bundles were collected from each colony.

Fertilisation and larval culture followed standard methods^18,43^. Gamete bundles were gently agitated to facilitate separation of sperm and eggs to maximise cross-fertilisation^44^. After one hour, the buoyant eggs were carefully siphoned off and placed in a fresh container of filtered seawater, to remove excess sperm. This process was conducted three times, to prevent polyspermy and maintain water quality during larval culture^44^. The developing embryos were then transferred to 1,000 L rearing tanks and left undisturbed, with ~300,000 embryos in each tank (~0.3 larvae/mL). Prior to transfer to the rearing rearing tanks, five 100 mL subsamples of embryos were collected for examination under dissecting microscopes and it was determined that >90% of the eggs were fertilised. After 24 hours, gentle aeration was supplied and 50 L of new filtered seawater was added daily to replace water lost to evaporation and to help maintain water quality^17^.

### Effect of larval density on settlement and persistence of corals on tiles

To test the effect of larval density on settlement rates and colony persistence, single replicates of 30 larval densities ranging between 10–5,000 were selected for investigation. Previous testing of densities with 500–1,000 larvae per tile indicated settlement rates can range between 10–20%, consistent with 10–15% settlement observed in other *in situ* settlement studies^21^. We therefore chose ~5,000 larvae as the upper density limit in this study, assuming this would result in a maximum of ~1,000 initial settlers on the highest density tile (~3.3 settlers/cm^2^), which would quickly be subject to intraspecific competition^22^. Early in the morning on the 4^th^ day after spawning (14 May 2017) competent larvae were carefully filtered out of the larval culture tanks with a 60 µm plankton mesh sieve, then extracted with pipettes and counted in Bogorov trays under dissecting microscopes illuminated with LED cold light sources. Groups of larvae that were actively swimming and exhibiting searching behaviour were counted and placed into 30 plastic bowls each containing ~500 mL of filtered seawater corresponding with the preselected larval densities. Manual counting time was minimised to avoid compromising larval health in the concentrated high density samples, so the preselected densities were used as an approximate guide, and where the numbers of larvae counted differed slightly from the predetermined densities, the actual numbers were recorded and used in all subsequent analysis (see Supplementary Dataset 1). A total of 45,518 larvae were individually counted, with the highest density treatment containing 5,130 larvae. Larvae in the bowls were gently swirled every 1–2 hours to prevent early settlement behaviour. Shortly before transfer to the field, larvae were further concentrated into 30 labelled 60 mL syringes, each containing 60 mL of filtered seawater.

**Figure 1:**
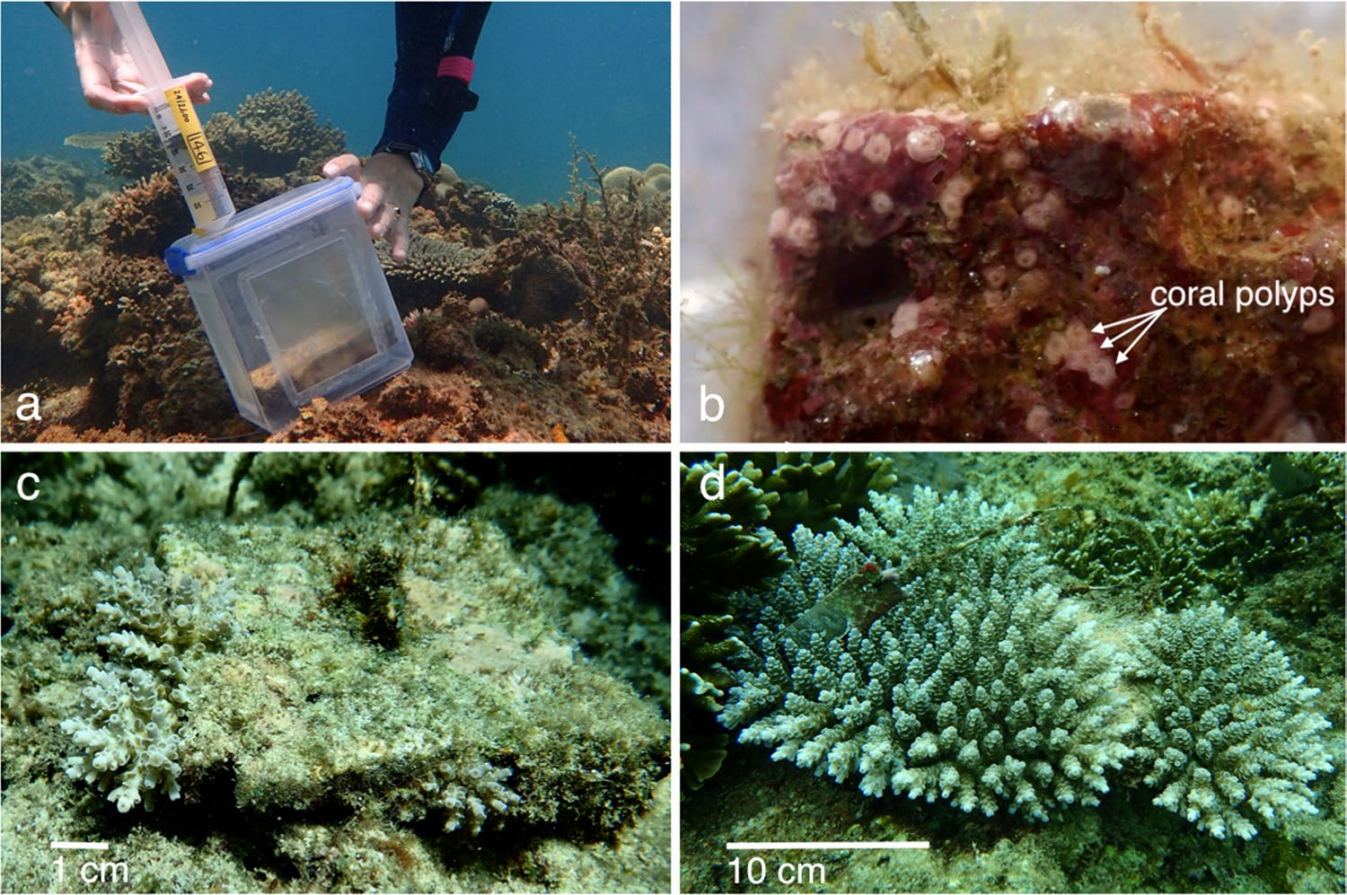
Images from the field site at Magsaysay Reef, Luzon, the Philippines: **(a**) injecting *A. tenuis* larvae into the flow-through containers at the Magsaysay Reef site with settlement tile visible within the container, (**b**) five day old spat on 18 May 2017, visible as small pink polyps, on a settlement tile, (**c**) juvenile corals surviving after 12 months (5 May 2018) on a tile initally supplied with 991 larvae, and (**d**) the same tile with surviving juvenile corals after 24 months (24 April 2019). Photos: (**a**) D. dela Cruz, (**b–d**) K. Cameron.

The sealed larval syringes were carefully transported from BML to Magsaysay Reef in a large plastic container filled with seawater to regulate temperature and provide gentle agitation to prevent larvae from settling in the syringes. Before release of larvae into settlement enclosures, each *in situ* tile was briefly removed from its post and visually checked for recent wild coral recruits. A small number of juvenile pocilloporid recruits were observed and removed, however no juvenile *Acropora* spp. recruits were found. Modified 2.3 L food quality polyethylene containers were used as the settlement containers for the field settlement experiment (Fig. 1a). Each container was threaded over a steel post, with 150 µm plankton mesh windows on each side to allow water exchange while containing the *A. tenuis* larvae, as these typically exceed 300 µm in diameter^18,45^. The settlement tile was replaced atop a rubber spacer to seal the post hole in the bottom of the container and the container lid was secured with an O-ring, creating a flow-through container around each tile, with the tile orientated in the same aspect in which it had been conditioning. Each container lid was pre-drilled with a small hole through which the counted larvae were injected (Fig. 1a), then the hole was sealed with a self-tapping screw.

The site was monitored daily for five days and all larval containers remained secure. After the five day settlement period, the larval containers were removed and tiles were carefully transported to a nearby field laboratory for examination under dissecting microscopes and LED lights, with the tiles remaining submerged in seawater in large containers. Tiles were initially examined under low magnification to confirm that no previously settled natural *Acropora* spp. recruits were present. None were detected, consistent with negligible recruitment observed for wild *Acropora* spat at Magsaysay Reef^18^. The position on the tile of each newly settled and metamorphosed coral spat was then recorded and scored as either top (upward facing surface), vertical edge, underhang (outer one cm of the downward facing surface) or inner bottom (inner eight cm of the downward facing surface). Each tile surface was also photographed (Fig. 1b). A thick, anoxic film of cyanobacteria had developed on the bottom and sides of the tile with the fourth highest larval density (2978 larvae supplied), rendering it unsuitable for any settlement, so data from this tile were discarded from analyses. The remaining 29 tiles were returned to the study site within several hours after collection, each to the same location and reattached onto the post in the same orientation as it was placed during conditioning and larval settlement.

Tiles were collected and surviving juvenile corals counted again under microscopes after two months (26 July 2017), five months (25 October 2017) and eight months (23 January 2018). At 12 months (5 May 2018, Fig. 1c) and 24 months (24 April 2019, Fig. 1d) the surviving corals were too large for tiles to be removed without damaging the coral colonies, so visual monitoring was completed by divers in the field. Counting individual colonies was challenging after 12 months, as fusing colonies became difficult to distinguish independently, so at 12 and 24 months, the greatest diameter (GD) and least diameter (LD) of each discrete colony or discrete collective of fused colonies was measured with calipers. The geometric mean diameter √ (GD × LD) was subsequently calculated for each colony^46^ or fused colony, and these were summed to derive the total horizontal area of coral growing on each tile.

### Statistical analyses

Data analyses were undertaken using the statistical package R, v.3.3.3^47^. Analyses of initial settlement and colony persistence to 12 months were conducted with generalised additive models (GAM) from the package ‘mgcv’^48^, using negative-binomial variance structure to account for overdispersion evident in the data. For each census period, the GAM included initial larval supply as the continuous predictor and the number of spat (5 days) or colonies present (2, 5, 8 and 12 months) as the response variable. Analysis of initial settlement rate was conducted with a GAM using binomial variance structure, with initial larval supply as the continuous predictor and the proportion of larvae settled as the response variable. Analysis of coral cover at 12 and 24 months was conducted with GAMs using normal variance structure, with initial larval supply as the continuous predictor, and cm^2^ of coral cover per tile and average colony size as the response variables. Comparisons of settlement on the available tile surfaces were initially conducted using a multivariate linear model, with initial larval density as the continuous predictor and the proportion (%) of settled larvae in three of the four settlement positions as response variables. Four univariate linear models were then fitted with the proportion of settled larvae in each settlement position as the response variable, and initial larval density as the continuous predictor. The appropriateness of all models was examined through visual inspection of residuals, and all figures were created using the ‘ggplot2’ package^49^ and ‘cowplot’ package^50^.

## Results

### Effect of larval supply density on initial settlement

**Figure 2:**
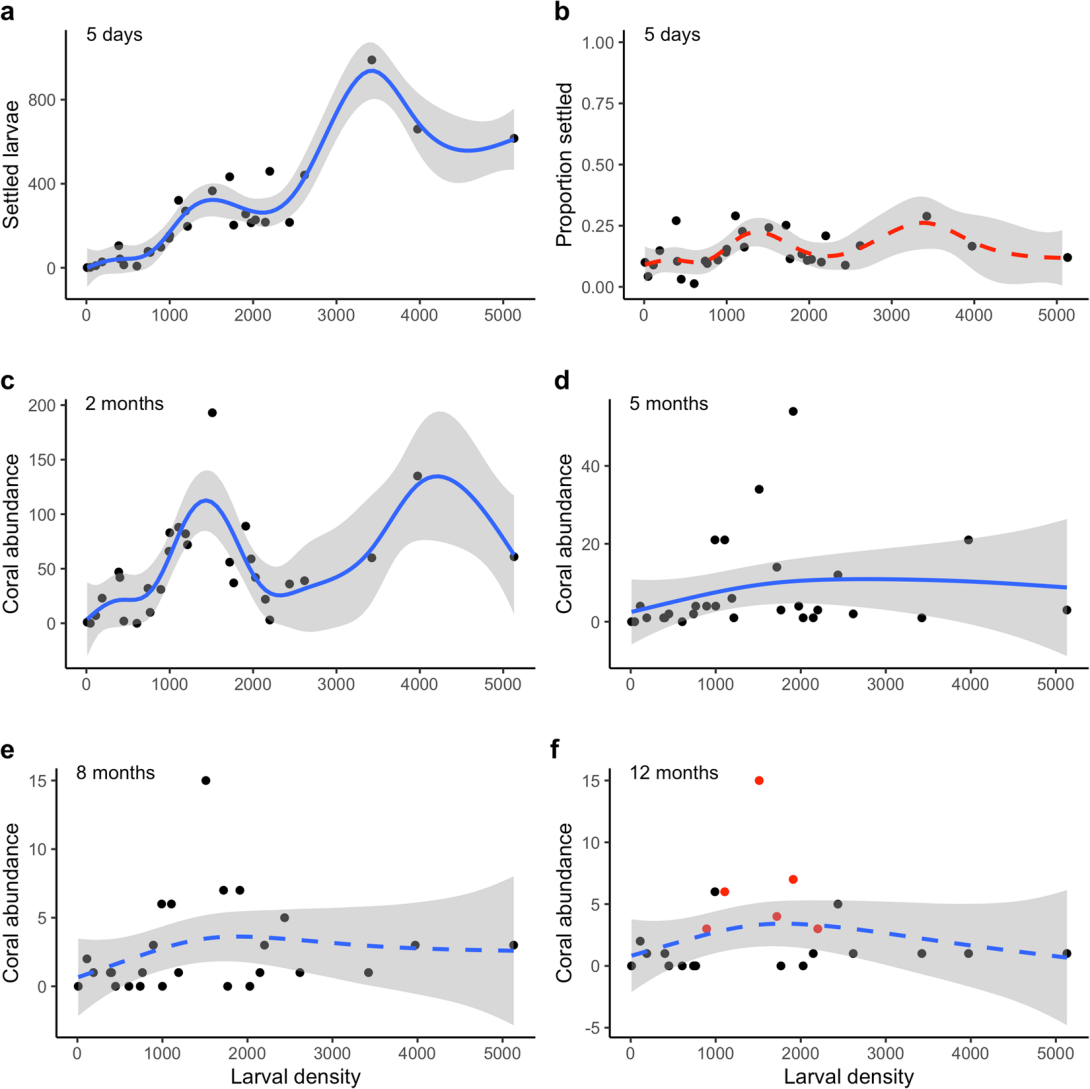
Relationship between larval density and initial settlement and colony abundance during the first 12 months: (**a**) total *A. tenuis* settlement after five days, (**b**) proportion of larvae settled (%) after five days, (**c**) post-settlement colony abundance after 2 months, (**d**) colony abundance after 5 months, (**e**) after 8 months, and (**f**) after 12 months, with visibly fusing colonies highlighted with red data points. Solid lines indicate significant model fits, dashed lines represent non-significant model fits, and shaded areas represent 95% confidence intervals.

From 42,540 larvae supplied, a total of 6,839 settled coral spat (16% total settlement) were counted on 29 tiles after the five day settlement period. A positive relationship was observed between larval supply and the number of settled coral spat (*p* = <0.001, R^2^ = 0.81, Fig. 2a and Table 1). However, rates of settlement (as a proportion of supplied larvae) were variable across the larval densities supplied, ranging between 3–30% with a mean of 14.4 ± 7.38% (s.d.) and showing no significant trend (Fig. 2b).

**Table 1:** Summary of generalised additive models testing the response of larval density on coral settlement, juvenile abundance and coral cover.

Census period	n (tiles)	Response variable	Totals	Est. degrees freedom	p-value (spline)	R^2^	Deviance explained
5 days	29	No. settled larvae	6839	*4.29*	<*0.001*	*0.81*	*77.4%*
2 months	29	No. discrete colonies	1417	*3.45*	*0.004*	*0.34*	*33.0%*
5 months	29	No. discrete colonies	222	*2.94*	*0.009*	*0.06*	*33.0%*
8 months	26	No. discrete colonies	61	2.22	0.219	0.05	18.1%
12 months	23	No. discrete colonies	58	2.30	0.182	0.11	24.3%
		Coral cover (cm^2^)	170 cm^2^	*7.42*	*0.013*	*0.74*	*84.1%*
24 months	21	Coral cover (cm^2^)	2049 cm^2^	2.51	0.23	0.47	57.0%

The census period and corresponding number of tiles available for examination are indicated, as well as statistical model details and outcomes. Italic text indicates effects with p < 0.05.

### Coral abundance to 12 months

**Figure 3:**
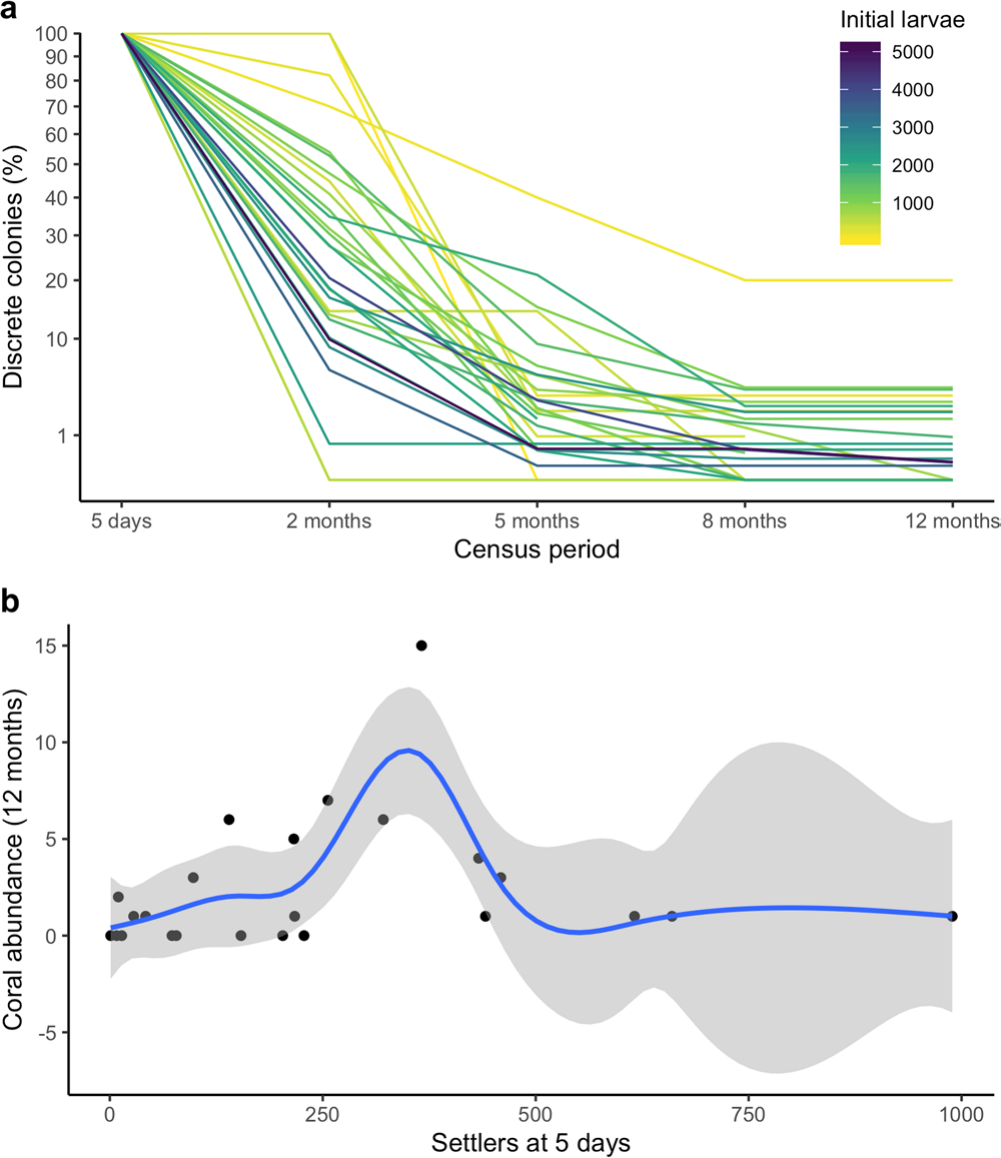
Changes in relative abundance of coral colonies during the first 12 months: **(a**) proportional change in number of colonies between census periods, and (**b**) relationship between the density of five day old settlers and colony abundance at 12 months. The solid line in (**b**) indicates significant model fit and shaded areas represent 95% confidence intervals.

Overall, steep declines in abundance of juvenile corals occurred on all tiles until five months after settlement, with continuing declines from five months to eight months and then negligible change in abundance from eight months to 12 months (Fig. 3a). It was not possible to consistently differentiate which individual corals were lost due to mortality or which fused with closely aggregated neighbours. Therefore, estimates of survival rates could not be quantified, and declines in numbers of observable juvenile colonies between census periods are presented as proportions (Fig. 3a). A significant non-linear relationship was found between settler density at five days and colony abundance at 12 months, with an optimum apparent at ~300 initial settlers, and only one colony per tile persisting to 12 months on tiles with more than 500 initial settlers (*p* = 0.06, R^2^ = 0.22, Fig. 3b). The total numbers of discrete juvenile corals counted on all tiles at each monitoring period to 12 months are shown in Table 1.

After two months, the relationship between coral abundance and initial larval density remained non-linear, with splines predicting a peak of ~50–100 corals on tiles from containers with ~1,000–2,000 initial larval densities (Fig. 2c). A second peak of greater than 100 corals was predicted for tiles with ~4,000 initial larval density, however this prediction appears strongly influenced by only a single tile within the larval density range of ~3,500–5,000. After five months (Fig. 2d), the non-linear response to initial larval density had simplified to a single shallow inverted curve and high coral abundances were no longer predicted from tiles with the highest larval densities. At eight (Fig. 2e) and 12 months (Fig. 2f), the response of coral abundance to larval density was no longer significant due to high variance in numbers of coral colonies on tiles treated with larval densities ranging between ~1,000–2,500. However, the trend of low coral abundance at the lowest and highest larval densities remained, with only tiles initially supplied with ~1,000–2,000 supporting more than five coral colonies. A single tile supplied with 1,511 larvae was found with 15 surviving juvenile corals at eight and 12 months, more than double that of any other tile. This tile is clearly discernable in Fig. 2e,f. There were no grounds for its exclusion as an outlier, and its removal did not change the shape of the trends. Some degree of fusion of close neighbour juvenile corals was clearly visible on six tiles at 12 months, highlighted as red data points in Fig. 2f. These tiles were initially supplied with ~900–2,200 larvae.

At the eight month monitoring period, three tiles were found smothered by a fast-growing encrusting *Montipora* coral spp. from the adjacent reef (see Supplementary Fig. S3). While all tiles were subject to competition with other reef organisms, the *Montipora* overgrowth interactions were particularly rapid and comprehensive. Therefore, these tiles were removed from analysis at the point they became overgrown, as the resultant mortality was clearly attributable to a strong pressure not acting on other tiles. One more tile was overgrown at 12 months, when two other tiles became dislodged and were smothered by sand. By 24 months, two more tiles had became dislodged and were removed from analysis, leaving 21 tiles in the experiment at 24 months (Table 1).

### Coral growth at 12 months and 24 months

**Figure 4:**
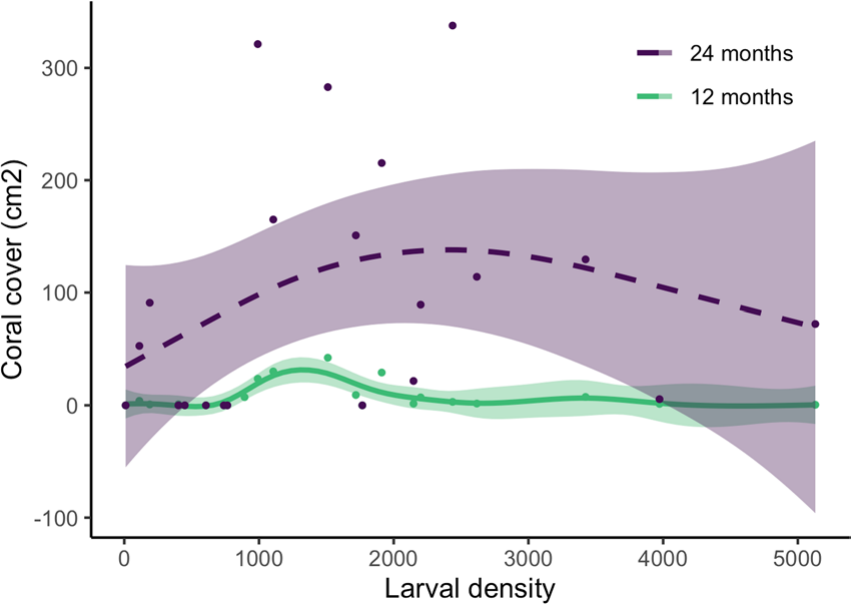
Comparison of live coral cover contributed by coral colonies on each tile at 12 months and 24 months. The solid line for 12 months indicates significant model fit, the dashed line for 24 months represents non-significant model fit, and shaded areas represent 95% confidence intervals.

With observable fusion of colonies occurring on some tiles at 12 months, the area of live coral cover on each tile became a more appropriate measure of coral abundance between 12–24 months than individual colony counts. At 12 months, an optimum for coral cover was predicted for tiles with ~1,500 initial larval density (*p* = 0.06, R^2^ 0.16, Fig. 4, Table 1), with tiles initially supplied with ~1,000–2,000 larvae having the greatest coral cover (>20 cm^2^). This trend had lessened by 24 months due to high variance in the response data and was no longer significant (Fig. 4, Table 1). However, tiles with the highest coral cover (>120 cm^2^) were those initially supplied with ~1,000–2,500 larvae, with lower coral cover on tiles with initial larval densities of less than ~1,000 and greater than ~3,000.

### Effect of larval supply density on settlement choices

**Figure 5:**
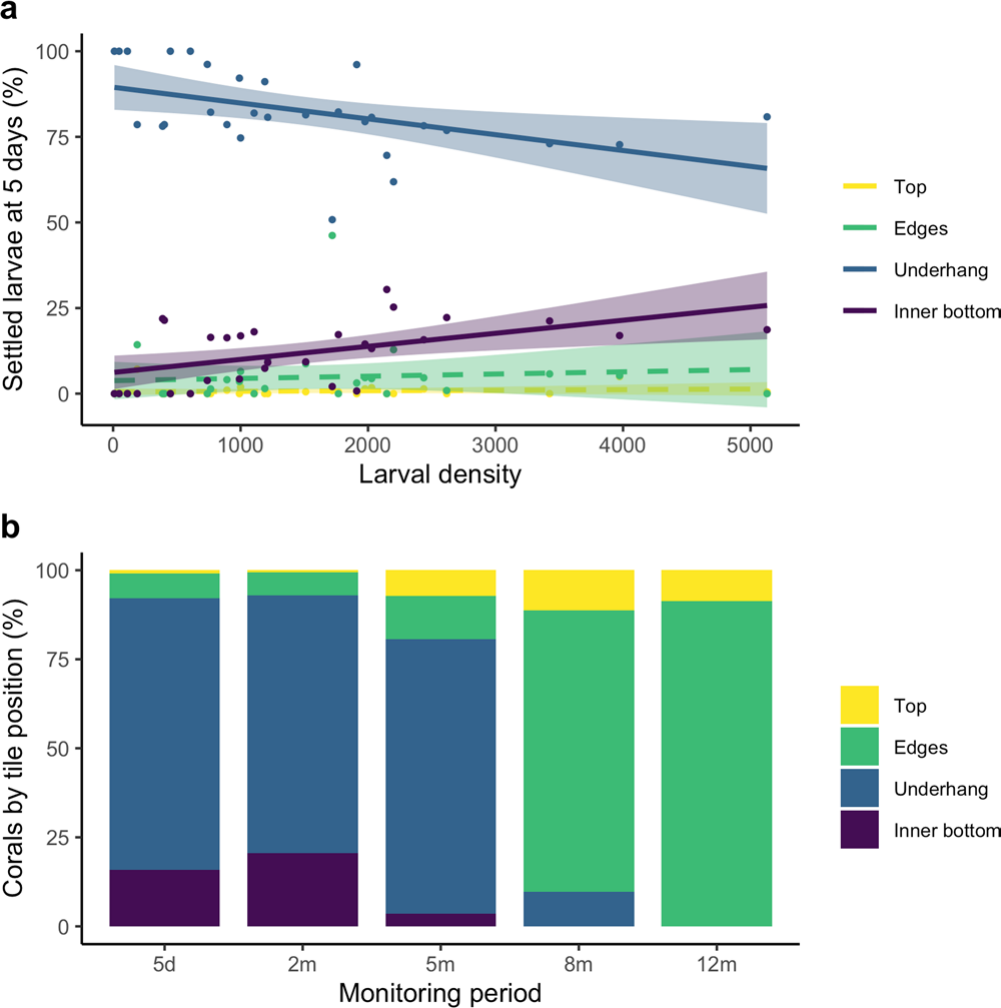
Positions of coral spat and corals on tiles: (**a**) initial settlement after five days of *A. tenuis* in available tile positions under the range of larval densities investigated, and (**b**) settlement and abundance of corals on all tile positions from five days to 12 months. In (**a**), solid lines indicate significant model fits, dashed lines represent non-significant model fits, and shaded areas represent 95% confidence intervals.

Increasing larval density had a significant effect on the settlement positions chosen by larvae (multivariate linear model: *F* = 3.61, *p* = 0.027, R^2^ 0.30), monitored at five days after larvae were supplied to the tiles and measured as proportions of settled larvae (i.e., excluding larvae that did not settle). At the lowest larval densities, almost all settlement was on the underhang position of the tiles, however this proportion declined as larval density increased (*F* = 7.5, *p* = 0.01, R^2^ = 0.22, Fig. 5a, Table 2). Declines in proportional settlement on underhangs correlated with increasing settlement on the inner bottom of the tiles as larval density increased (*F* = 9.3, *p* = 0.005, R^2^ = 0.26, Fig. 5a, Table 2). Settlement on the top and vertical edges of the tiles was very low and not affected significantly by larval density (Fig. 5a, Table 2). Over 90% of initial larval settlement and of corals present at two months were in the downward facing surfaces of all tiles, with 76.4% of settlement in underhang positions (Fig. 5b). However, 91.4% of the corals present at 12 months were recorded as growing on the vertical edges of tiles (Fig. 5b). More than half of these corals had originally settled in underhang positions, then grown upwards through asexual budding and colony growth to the vertical edges.

**Table 2:** Summary of linear models testing the effect of larval density on the per cent of settled larvae in each of four settlement positions.

Settlement position (% settled larvae)	Direction	*F*-statistic	Std error	*p* value	R^2^
Top	~	0.43	144.30	0.52	0.02
Edge	~	0.21	26.11	0.65	0.01
Underhang	−	*7.5*	*17.22*	*0.01*	*0.22*
Inner bottom	+	*9.3*	*22.02*	*0.005*	*0.26*

The directions of effect are indicated as well as statistical model outcomes. ~, no relationship; −, negative relationship; +, positive relationship. Italic text indicates effects with *p* < 0.05.

## Discussion

Determining optimal supply densities of larvae to enable robust settlement and persistence of juvenile corals on degraded reef substrata is an important issue for coral reef restoration^6,18^, as density-dependent effects are known to influence early stages of coral recruitment^12,17,21^. This study provides the first *in situ* evaluation of larval supply density on settlement and juvenile colony persistence on tiles over an extended monitoring period of two years, undertaken entirely at a natural, degraded coral reef.

**Figure 6:**
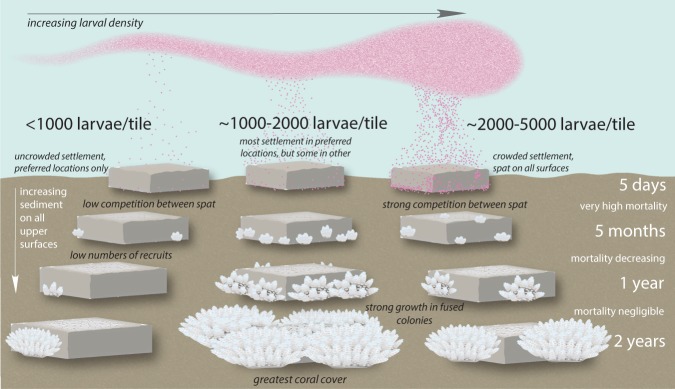
A conceptual diagram showing the relationship between larval density and initial settlement, colony abundance to one year, and overall live coral cover to two years on *in situ* settlement tiles. Illustration by K. Cameron.

Consistent with other studies, we found a strong positive relationship between larval density and total larval settlement, and also that post-settlement persistence of juvenile corals was lowest on tiles with the highest larval densities^12,17,21^. Our experiment extended these previous findings by investigating effects of a greater range of larval densities over a longer period. We found that from five to 24 months after settlement, colony abundance and later coral cover remained lower on high larval density tiles (>~2,500 larvae) compared with lower mid-range larval density tiles (~1,000–2,500 larvae). We suggest this indicates the larval saturation point in this experiment where density-dependent mortality may have overridden the positive correlation between settlement and recruitment^51^, while the lower-mid range densities maximised the persistence of corals through their critical first six months when post-settlement mortality is very high^18,23,24^. Overall, optimal supply density for *A. tenuis* to maximise coral abundance to 12 months under these *in situ* experimental conditions was ~1,500 larvae per tile (5 larvae cm^−2^), with a range of ~1,000–2,000 larvae per tile (~3.3–6.7 larvae cm^−2^) generally providing both consistent settlement and post-settlement abundance. The relationship between larval density, settlement, post settlement colony abundance and coral cover is summarised in a conceptual diagram (Fig. 6).

A novel finding from this study was the non-linear relationship between initial larval density and coral growth, an important consideration for coral restoration efforts aiming to facilitate rapid recovery of degraded reefs. We found significantly less coral cover on high larval density tiles compared to mid-range density tiles after 12 months (Fig. 4). The relationship between larval density and coral cover was no longer significant after 24 months, as variance in colony growth increased over time. However, the general trend persisted, with higher coral cover on mid-range larval density tiles than on high density tiles after 24 months, resulting from greater outgrowth of colonies from the mid-range tiles between the 12 and 24 month monitoring periods (Fig. 1d). The lower coral cover on the highest density tiles at 12 months and 24 months suggests that high intraspecific competition could potentially cause negative outcomes for larval restoration on degraded coral reefs if larval densities are too high.

Competition for space as juvenile corals grow is a significant factor affecting adult survival^22,32^. Closely aggregated spat may either have a single genotype dominate and thrive while others perish, or individual colonies may fuse to become chimeric colonies^12,18^, particularly during the first 8–10 months after settlement and when relatedness between colonies is high^38,39^. Chimerism has been reported in *Acropora* species, following dense settlement aggregations under experimental conditions, and can lead to rapid growth and early onset of sexual maturity^12,18,39^. Fusion of juvenile corals was clearly observed on six of our study tiles at 12 months post-settlement (Fig. 2f), and probably commenced earlier although was not visible under field monitoring conditions. The tiles with obvious fusion after 12 months were those with the highest number of juvenile corals, initially supplied with ~1,000–2,000 larvae and also supporting the highest coral cover at 12 months. Increases in coral cover varied greatly between 12 to 24 months and the factors influencing differences in growth rates require further study, particularly genetic analyses to determine whether chimerism may have contributed to the rapid growth of the large colonies^38^. Enhancing larval densities amplifies opportunities for gregarious settlement^18^, therefore larval restoration of degraded coral reefs using appropriate larval densities could also support development of chimeric colonies with the potential to grow rapidly^38^.

To uncouple the influence of initial settlement on post-settlement survival trends, we considered the effect of settler density on colony persistence and found settler densities of ~300 coral spat/tile (~1.0 spat cm^−2^) generally yielded the highest colony abundance at 12 months (Fig. 3b). This is double the settler density recommended by Suzuki *et al*.^21^, who found survival to one month on settlement grid plates with 0.5 spat cm^−2^ was much less than with lower settler densities (<0.1 spat cm^−2^) in a field experiment in the Ryukyu Archipelago, Japan. However, the Ryukyu colonies experienced near total mortality between three to six months, which was attributed to high intraspecific competition and negative impacts from sedimentation and unstable fixtures to the reef, after which the experiment was concluded^21^. The potential impacts of sedimentation and stability of fixtures were mitigated in our study through careful attachment of tiles onto steel posts directly into hard reef substratum away from sand (although three tiles became dislodged and their corals quickly died). The contrast between our results on optimal initial settler density and those in the Ryukyu Archipelago^21^ indicates that density dependent mortality is part of a suite of selection pressures acting on early life-history stages of juvenile corals^10,15^. Where external pressures such as sedimentation and substratum stability can be managed and mitigated, higher settler densities can be beneficial, such as the settler densities of 0.5–1.5 spat cm^−2^ recommended for settlement on artificial subtrata suggested in coral restoration guidelines^37^.

Juvenile corals face significant survivorship bottlenecks during the early stages of development^12,17,21^, with very high mortality in the first six months post-settlement^18,23,24^. Our results followed this pattern, with very steep declines in abundance (>98%) observed on all tiles from settlement to 8 months, and negligible loss of corals from 8 months to two years. The potential impact of predation, limited light availability and sedimentation on post-settlement mortality can be either mitigated or increased by the orientation of the surface position chosen by coral larvae for settlement. Of the 61 colonies persisting on our tiles until 8 months, over 80% began as spat that initially settled on a vertical edge or underhang tile surface. Settlement in these semi-sheltered locations would have provided good access to light for photosynthesis^34^ and only limited sedimentation^52^, with underhang locations potentially having some protection from predation by corallivorous fish^53^. In contrast, corals that settled on upward facing surfaces would have had abundant light but also higher sedimentation and possibly increased risk of predation, while corals settled on inner bottom surfaces may not have thrived due to insufficient light.

Larvae in all densities examined showed a strong preference for settling in the advantageous underhang locations, however this was significantly negatively affected by increasing larval density (Fig. 5a). Highest settlement on inner bottom surfaces was found on the highest larval density tiles (<~2,500 larvae). Average settler density in underhang positions on these tiles was very crowded at 18.2 ± 5.66 spat cm^−2^ (mean ± s.d.), compared to average underhang settler densities of 7.5 ± 2.12 spat cm^−2^ (mean ± s.d.) on tiles with larval densities between ~1,000–2,000. Avoiding overcrowding may be a trade-off with advantageous substratum selection at very high larval densities, which may subsequently contribute to high post-settlement mortality in these densities.

Our results highlight the importance of carefully evaluating larval density when considering larval restoration methods for degraded coral reefs. While we set out to investigate whether an optimum larval density could be determined to minimise potential wastage of larvae, we found that supply of larvae at very high densities was not only inefficient, but could also have negative outcomes on subsequent colony survival and coral cover. We therefore suggest that larval densities from the lower end of the optimal range found in this study would be a logical approach for larval restoration of degraded reefs with *A. tenuis*. These densities lead to appropriate settler densities that did not impede larval preference for substratum selection, and with a high likelihood of colonies persisting to two years, including formation of rapidly growing fused colonies (Fig. 6). Further field tests to determine optimal larval supply densities in other species and locations are needed to continue exploring these parameters and optimise methods for larger-scale larval restoration of coral populations and co™mmunities.

## Supplementary information

Supplementary Figures.

Dataset 1.

## Data Availability

All data generated or analysed during this study are included in this published article. The data reported in this paper are included at Supplementary Dataset 1.
