# Supplementary material for: Density of coral larvae can influence settlement, post-settlement colony abundance and coral cover in larval restoration
Source: Sci Rep. 2020 Mar 26;10:5488. doi: 10.1038/s41598-020-62366-4 (PMC7099096; doi:10.1038/s41598-020-62366-4)

**Density of coral larvae can influence settlement, post-settlement colony abundance and coral cover in larval restoration**

**Kerry A. Cameron^a^* & Peter L. Harrison^a^**

^a^Marine Ecology Research Centre, School of Environment, Science and Engineering, Southern Cross University, Lismore, New South Wales 2480, Australia.

*K. A. C. email: [kerry.cameron@live.com.au](mailto:kerry.cameron@live.com.au)

**Figure S-1.** Settlement tile attached to natural reef substratum. Photo: K. Cameron.


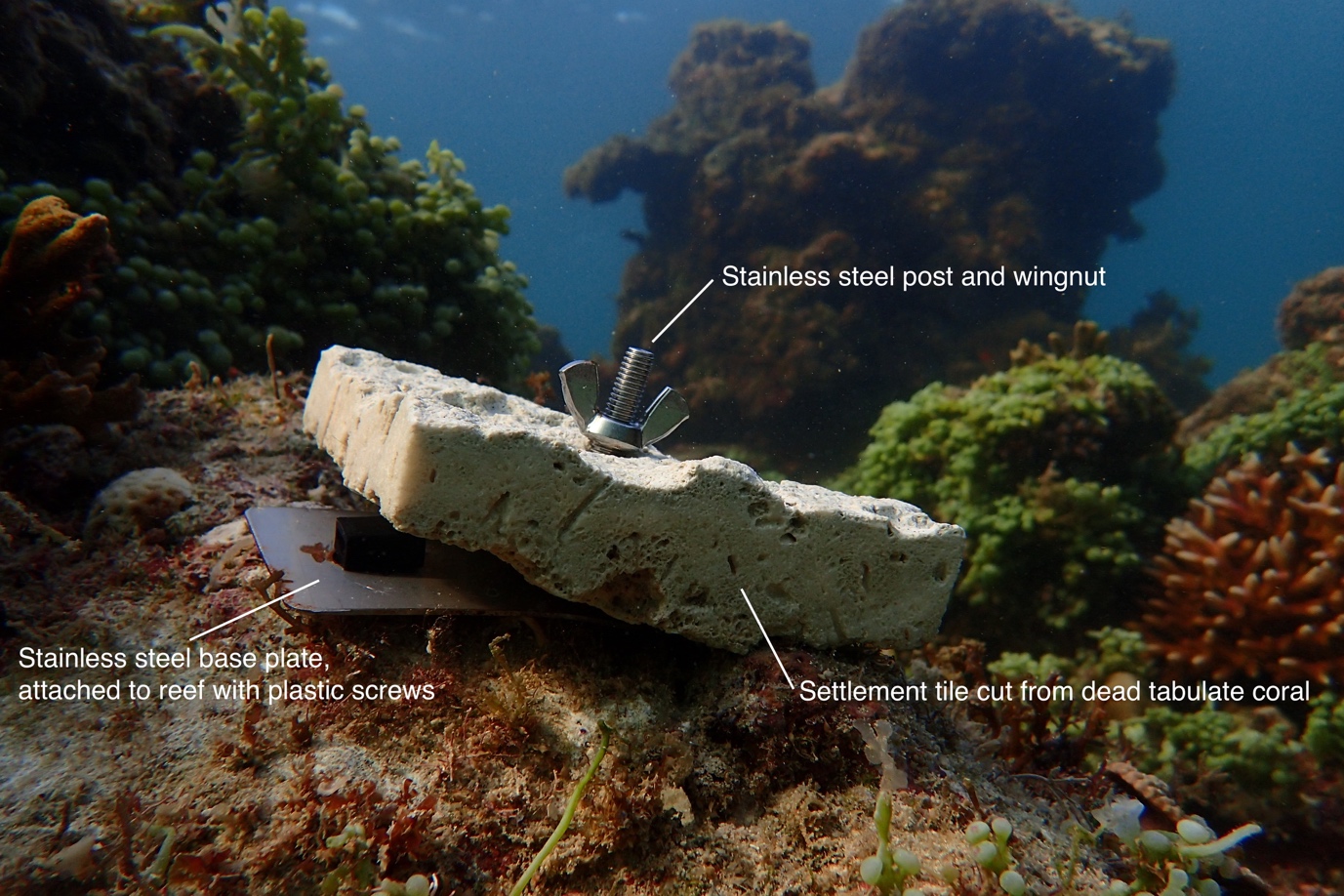


**Figure S-2.** Coral spawn collection nets: (a) collection nets placed over gravid *A. tenuis* colonies at dusk, each net weighted with a stainless steel ring at the base and kept upright with a small amount of air in each collection jar, and (b) buoyant gamete bundles floating up into the collection jar at the top of the net during coral spawning. Photos: K. Cameron.


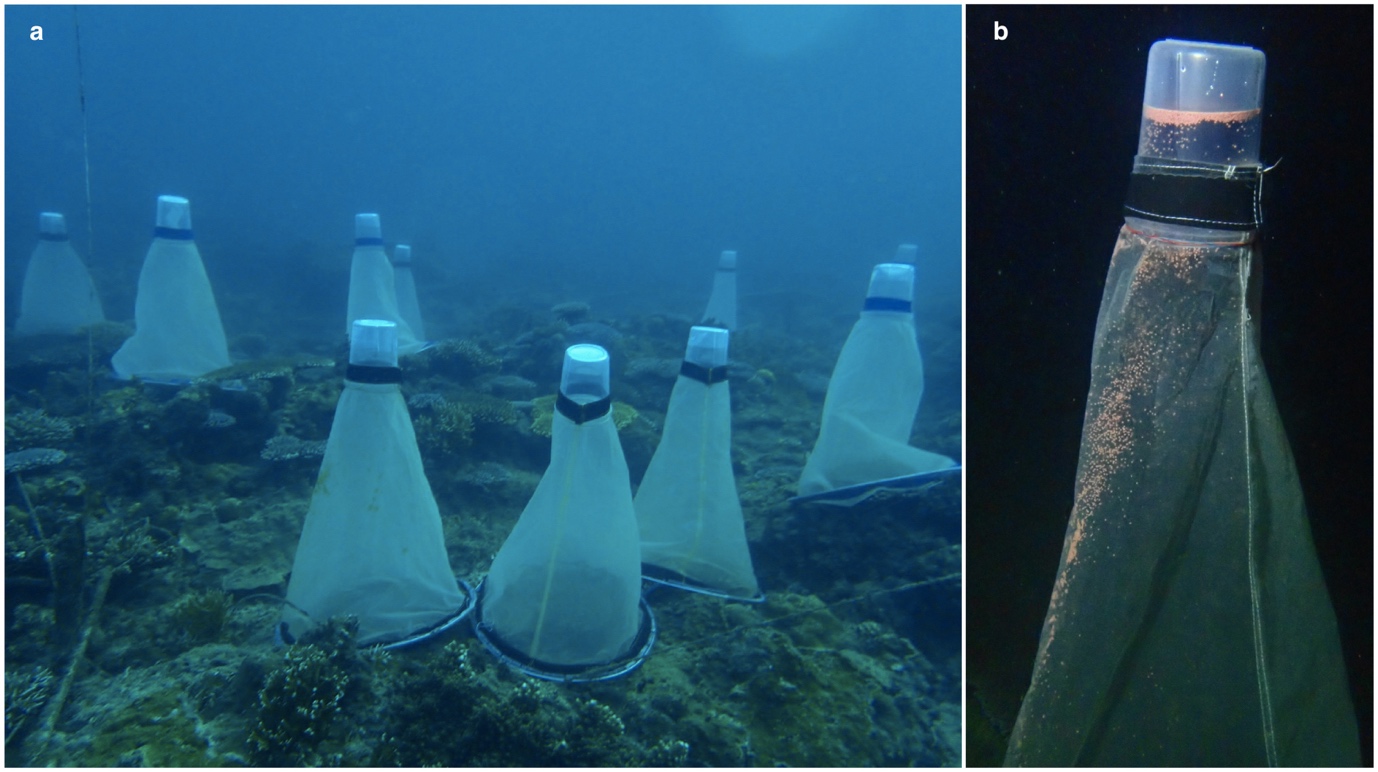


**Figure S-3.** Overgrowth of a settlement tile by *Montipora* spp. Four tiles were removed from analysis at the point they became completely overgrown (three tiles after eight months and one tile after 12 months). Photo: K. Cameron.


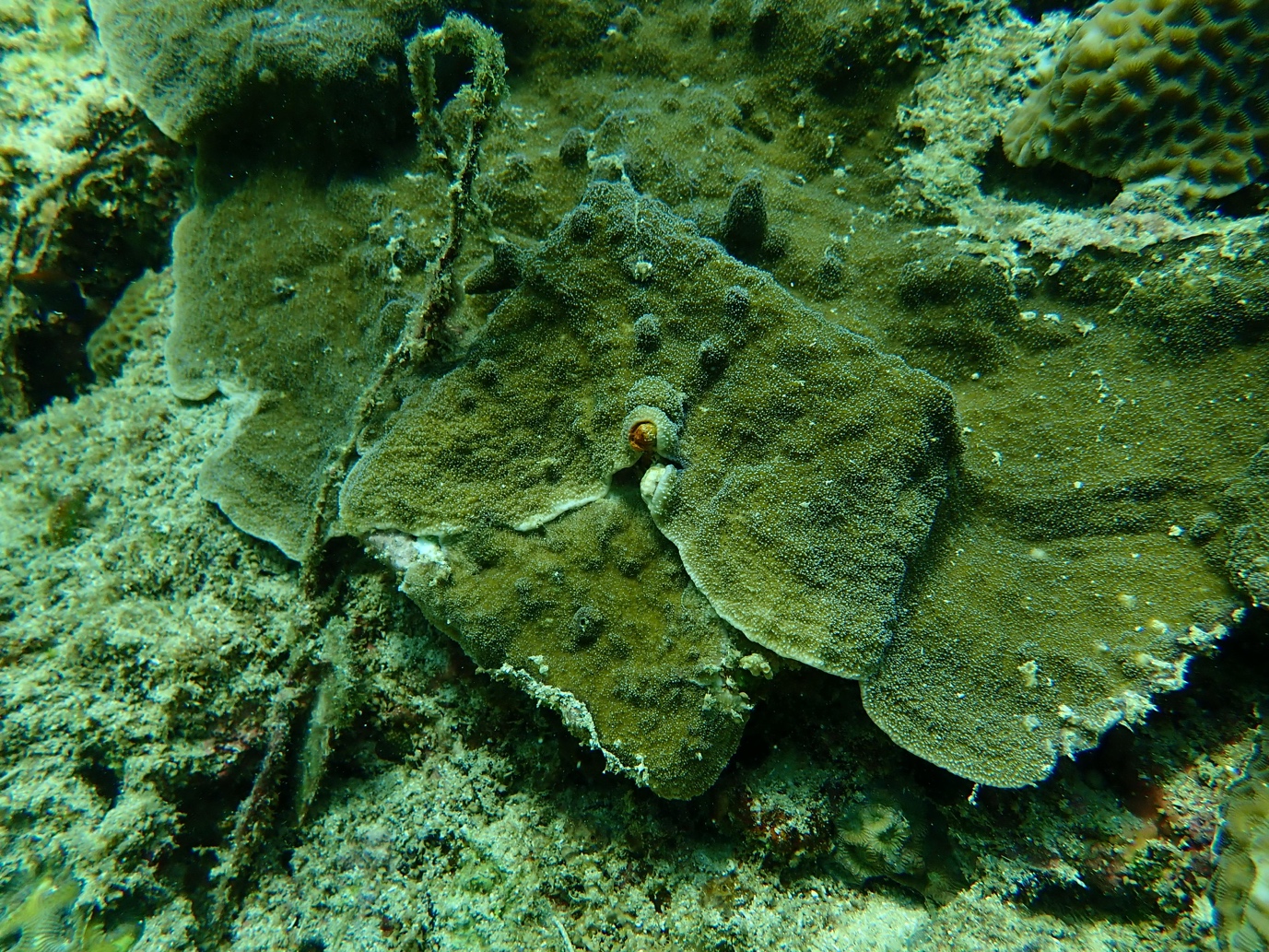

Supplement: Supplementary file 1 — Supplementary Figures. [file 41598_2020_62366_MOESM1_ESM.docx]
